# Development of a keratinase activity assay using recombinant chicken feather keratin substrates

**DOI:** 10.1371/journal.pone.0172712

**Published:** 2017-02-23

**Authors:** Hyeon-Su Jin, Seon Yeong Park, Kyungmin Kim, Yong-Jik Lee, Gae-Won Nam, Nam Joo Kang, Dong-Woo Lee

**Affiliations:** 1 School of Applied Biosciences, Kyungpook National University, Daegu, South Korea; 2 Department of Cosmetic Science & Technology, Seowon University, Cheongju, South Korea; 3 School of Food Science and Biotechnology, Kyungpook National University, Daegu, South Korea; Uniwersytet Gdanski, POLAND

## Abstract

Poultry feathers consist mainly of the protein keratin, which is rich in β-pleated sheets and consequently resistant to proteolysis. Although many keratinases have been identified, the reasons for their substrate specificity towards β-keratin remain unclear due to difficulties in preparing a soluble feather keratin substrate for use in activity assays. In the present study, we overexpressed *Gallus gallus* chromosomes 2 and 27 β-keratin-encoding genes in *Escherichia coli*, purified denatured recombinant proteins by Ni^2+^ affinity chromatography, and refolded by stepwise dialysis to yield soluble keratins. To assess the keratinolytic activity, we compared the proteolytic activity of crude extracts from the feather- degrading bacterium *Fervidobacterium islandicum* AW-1 with proteinase K, trypsin, and papain using purified recombinant keratin and casein as substrates. All tested proteases showed strong proteolytic activities for casein, whereas only *F*. *islandicum* AW-1 crude extracts and proteinase K exhibited pronounced keratinolytic activity for the recombinant keratin. Moreover, LC-MS/MS analysis of keratin hydrolysates allowed us to predict the P1 sites of keratinolytic enzymes in the *F*. *islandicum* AW-1 extracts, thereby qualifying and quantifying the extent of keratinolysis. The soluble keratin-based assay has clear therapeutic and industrial potential for the development of a high-throughput screening system for proteases hydrolyzing disease-related protein aggregates, as well as mechanically resilient keratin-based polymers.

## Introduction

Keratin is an insoluble, sulfur-containing fibrous protein and the main constituent of skin, hair, nails, hooves, horns, scales, claws, and teeth. It is synthesized by keratinocytes and is resistant to degradation by general proteases [1]. Based on their sulfur content, keratins can be divided into soft keratin (<10% cysteine) found in the epidermis of skin, and hard keratin (~10−14% cysteine) found in hair, nails, feathers, and claws [2]. The polypeptide chains of keratin are arranged into fibrous structures built from α-helices (α-keratin) or pleated β-sheets (β-keratins) held together by hydrogen (H) bonds and disulfide crosslinks [3]. This supramolecular architecture provides keratin with extraordinary rigidity. Poultry feathers comprise >90% keratin [4]. Global consumption of poultry meat is increasing, concomitant with an increase in unwanted poultry feathers as solid waste. This recalcitrant keratin biomass can be further hydrolyzed by chemical processes [5] to produce fertilizers, animal feedstock, and soil conditioner [6, 7]. The efficient conversion of feather keratin into soluble peptides is attractive for generating valuable products such as cosmetics [8], inexpensive and biodegradable thermoplastics [9], and construction materials [10]. Keratinase enzymes degrade the compact keratin materials, and these are distinct from more typical proteases. Understanding the nature of the efficient degradation of keratin by keratinases is therefore industrially and environmentally important.

Much effort has focused on the isolation and characterization of bacteria that degrade poultry feathers and human hair [11] (Table 1). Many microorganisms are able to degrade native chicken feathers to release free amino acids, particularly methionine and lysine [12–14]. In addition, putative keratinase-encoding genes from *Bacillus licheniformis* [15] *Fervidobacterium pennivorans* [16], *Streptomyces pactum* DSM 40530 [17], *Fumigatus fresenius* [18], *Trichophyton mentagrophytes* [19], and *Thermoactinomyces* sp. CDF [20] have been cloned, expressed, and characterized in detail. Extremophiles are an alternative source of enzymes for processing keratin waste at elevated temperatures [3, 21, 22]. Previously, we isolated and characterized the extremely thermophilic eubacterium *Fervidobacterium islandicum* AW-1, which could degrade native chicken feathers completely at 70°C under anaerobic conditions [14]. The near-complete genome sequence of *F*. *islandicum* AW-1 revealed a genome containing over 50 different proteases, of which some are presumably keratinolytic [23]. For instance, a novel type of M32 protease from *F*. *islandicum* AW-1 enhanced native feather degradation synergistically with crude extracts [24]. Nevertheless, there remain barriers to investigating the substrate specificity of keratinases and determining their kinetic parameters using insoluble feather keratins and keratin-like derivatives such as azo-keratin [25], keratin azure [26], and ball-milled feather powder [27]. A keratinolytic assay with an appropriate and soluble intact substrate is needed for a full characterization of keratinases, including assessment of their P1 sites.

**Table 1:** Bacterial and fungal keratinolytic enzymes.

Microorganism	Enzyme	Substrates	Temp. (°C)	pH	Unit	Ref.
*Bacillus licheniformis* PWD1	PE	Azokeratin	50	7.5	ΔA_450_ = 0.01	[25]
*Fervidobacterium pennavorans*	PE	Native feather meal	80	9.0	The residual dry weight of the remaining feather meal substrate	[21]
*Streptomyces* sp. S.K_1–02_	EE	Keratin azure	50	8.5	ΔA_595_ = 0.1	[26]
*B*. *subtilis* KS-1	EE	Azokeratin	30	7.5	ΔA_450_ = 0.001	[40]
*Thermoanaerobacter keratinophilus*	CE	feather meal	70	7.0	1 μmol of aromatic amino acids	[41]
*Stenotrophomonas* sp. D1.	EE	keratin powder	30	8.0	ΔA_660_ = 0.01	[42]
*Chryseobacterium* sp. kr6	CE	Azokeratin	50	8.0	ΔA_440_ = 0.01	[43]
*Microbacterium arborescens* kr 10	PE	Azokeratin	45	7.5	ΔA_420_ = 0.01	[44]
*B*. *subtilis* S 14	CE	Azokeratin	24	9.0	ΔA_450_ = 0.1	[45]
*B*. *subtilis* NRC 3	PE	aazokeratin	50	7.5	ΔA_450_ = 0.01	[46]
*Actinomadura keratinilytica* Cpt29	PE	keratin azure	70	10	ΔA_440_ = 0.1	[47]
*B*. *safensis* LAU 13	EE	feather powder	40	7.5	ΔA_280_ = 0.01	[48]
*B*. *pumilus* AT16	PE*	azokeratin	55	7.5	ΔA_450_ = 0.01	[49]
*Actinomadura viridilutea* DZ50	PE	keratin azure	80	11	ΔA_440_ = 0.01	[50]
*Thermoactinomyces* sp. RM4	EE	keratin azure	60	10.0	ΔA_595_ = 0.01	[51]
*B*. *subtilis* DP1	PE	chicken feather	37	10.0	increases absorbance by 0.1	[52]
*Caldicoprobacter algeriensis*	PE	keratin azure	50	7	ΔA_595_ = 0.01	[53]

PE, purified enzyme

CE, crude extract

EE, extracellular enzyme

*, recombinant

To this end, the aim of the present study was to develop a keratinolytic assay using soluble feather keratin as substrate and use this to biochemically and biophysically characterize keratinases from *F*. *islandicum* AW-1. The *Gallus gallus* genome is now available (Galgal 5.0), and genes FK4, FK12, and FK12 on chromosomes 2, 25, and 27, respectively, were found to encode β-keratins [28]. The *Gallus gallus* genome has revealed details of the genomic evolution, development, and differential expression of poultry keratin [29–31]. In the present work, we chemically synthesized cDNA genes encoding several feather β-keratins and expressed them in *Escherichia coli*. Subsequent characterization of *F*. *islandicum* AW-1 crude extracts with the recombinant feather keratins and casein as substrates was performed, and commercially available proteases proteinase K, trypsin, and papain were analyzed for comparison.

## Materials and methods

### Ethics statement

Chicken feathers as additional waste materials were obtained from a medium-sized poultry production unit in Kyungpook province. This study is not confined to the animal studies authorized by Institutional Animal Care and Use Committee at Kyungpook National University. No approval from an ethical committee was required for this study.

### Bacterial strains and culture conditions

*E*. *coli* DH5α and *E*. *coli* BL21 (DE3) (Novagen) were used for plasmid construction and protein expression, respectively. Cells were grown overnight in Luria-Bertani (LB) medium containing kanamycin (50 μg/ml) or ampicillin (100 μg/ml) in a rotary shaker at 37°C. For expression of recombinant keratin, *E*. *coli* BL21 (DE3) cells transformed with pET-28a(+)_Chr2_FK4, pET-28a(+)_Chr25_FK12, and pET-28a(+)_Chr27_FK12 were grown in LB medium (1 L) containing 50 μg/ml kanamycin at 37°C to an optical density at 600 nm of ~0.4−0.6. After induction with 1 mM isopropyl-β-D-thiogalactopyranoside (IPTG), cells were cultured for an additional 16 h and harvested by centrifugation (10,000 × g, 20 min, 4°C). Bacterial pellets were stored at -70°C until needed. Growth was monitored by determining the absorbance at 600 nm with an Ultraspec 8000 spectrophotometer (GE healthcare, PA, USA).

*F*. *islandicum* AW-1 (KCTC 4680) cells were grown in a modified *Thermotoga-Fervidobacterium* (mTF) medium supplemented with (per L) 0.1 g of NH_4_Cl, 0.16 g of MgSO_4_·7H_2_O, 0.9 g of NaH_2_PO_4_·2H_2_O, 1.6 g of K_2_HPO_4_, 1.0 g of yeast extract, 1.0 mg of resazurin, 0.8% (w/v) feather or 0.5% (w/v) glucose, 10 ml of a trace element solution (DSM medium 141), 10 ml of a vitamin solution (DSM medium 141), and 3 ml of 25% Na_2_S·9H_2_O. Cultures were grown at 70°C in sealed serum bottles under N_2_ gas. Chicken feathers were washed with deionized water to remove unwanted materials such as skin and dust, air dried at room temperature to remove moisture, and used as substrate in batch feather degradation experiments.

### Construction of FK genes encoding feather keratins

A search of the *Gallus gallus* 5.0 chromosome sequences in GenBank identified, putative feather keratin (FK) genes on chromosomes 2 (CM000094.3), 25 (CM000124.4), and 27 (CM000118.4). Alignments of keratin amino acid sequences were performed using Clustal-W, and phylogenetic trees were built with MEGA7 [32]. To construct FK expression vectors, codon-optimized Chr2_FK4, Chr25_FK12, and Chr27_FK12 genes with overhanging *Nde*Ι and *Xho*Ι sites were synthesized by Bioneer Co. (Daejeon, Korea) and cloned into the pBHA vector to yield plasmids pBHA-Chr2_FK4, pBHA-Chr25_FK12, and pBHA-Chr27_FK12. These plasmids were transformed into *E*. *coli* DH5α competent cells, and transformants containing the pBHA vectors harboring the FK genes encoding *Gallus gallus* keratins were selected on LB medium-ampicillin plates. Plasmids were isolated from the transformants and digested with *Nde*I and *Xho*I. Inserts were purified and ligated into the *Nde*I and *Xho*I sites of the pET-28a (+) plasmid (Novagen) to yield pET-28a_Chr2, pET-28a_Chr25, and pET-28a_Chr27. Expression vectors also encoded an N-terminal polyhistidine (×6His) tag in frame with the inserted gene.

### Purification and refolding of feather keratins

Centrifuged cells were suspended in lysis buffer (50 mM NaH_2_PO_4_, 300 mM NaCl, 10 mM imidazole, 1 mM PMSF, pH 8.0) and disrupted by sonication (2 s pulse with 5 s pause for a total period of 40 min). Lysates were centrifuged at 10,000 × g for 30 min to collect cell debris including expressed keratins as inclusion bodies, and supernatants were discarded. Cell pellets were resuspended in lysis buffer containing 8 M urea, incubated on ice for 1 h to completely dissolve protein, and centrifuged at 16,000 × g for 30 min. The resulting supernatants were filtered through a 0.45 μm filter, and filtrates were loaded on a Ni-NTA agarose resin (Qiagen, Germany) column (10 ml) equilibrated with 8 M urea containing lysis buffer according to the manufacturer's instructions. Briefly, the column was washed with 16 column volumes of the same buffer, and 250 mM imidazole was applied to elute recombinant proteins. Eluents containing unfolded keratin were concentrated using a centrifugal concentrator with a 3,000 (Millipore, USA), and buffer-exchanged by step-wise dialysis against 50 mM Tris-HCl (pH 8.0) at 4°C. Dialyzed samples were centrifuged at 10,000 × g for 30 min to remove insoluble material, and the resulting supernatants containing refolded keratin were concentrated using a centrifugal concentrator with a 3,000 MWCO membrane (Millipore) and stored at 4°C until needed. The protein concentration was determined by the bicinchoninic acid (BCA) assay [33] with bovine serum albumin as a standard. In addition, the concentration of purified keratins was determined using individual extinction coefficients obtained from experimental data. Enzyme fractions were analyzed by 12% sodium dodecyl sulfate polyacrylamide gel electrophoresis (SDS-PAGE) and visualized with Coomassie blue staining [34].

### Preparation of crude extracts from *F*. *islandicum* AW-1

*F*. *islandicum* AW-1 cells grown in mTF medium containing 0.5% (w/v) glucose at 70°C for 12 h were used to inoculate freshly prepared mTF medium supplemented with 0.8% (w/v) native chicken feathers. After 10 h of anaerobic incubation, the culture medium was filtered through a No. 20 filter paper (5−8 μm cutoff, Hyundai, Korea) under vacuum to remove residual chicken feathers, and cells were harvested by centrifugation at 10,000 × g for 20 min at 4°C. Cell pellets were washed twice with 50 mM HEPES buffer (pH 8.0) and disrupted by sonication on ice for 5 min (2 s pulse with 5 s pause at a power setting of 30%). Cell debris was removed by centrifugation at 10,000 × g for 20 min at 4°C, and the resulting supernatant, defined as crude extract (AWCE), was used for further experiments.

### Preparation of fluorescently labeled keratin substrates

Fluorescein-5-maleimide (FM; Molecular Probes Inc., Eugene, OR, USA) was freshly prepared as a stock solution in 10 mM dimethyl formamide (DMF) in the dark. Purified recombinant keratins were reduced by the addition of a 10-fold molar excess of Tris (2-carboxyethyl) phosphine (TCEP) in 50 mM Tris buffer (pH 7.0) for 30 min, then alkylated with a 25-fold molar excess of FM for 2 h. Thereafter, the conjugate was loaded on a Superdex 200 10/300 GL column (GE Healthcare, PA, USA), and labeled protein was stored at 4°C until needed.

### Enzyme activity assay

Caseins from bovine milk and papain were purchased from Sigma-Aldrich (St. Louis, MO, USA), and proteinase K and trypsin (powder form) were from Promega (WI, USA). For proteolytic activity assays, we modified the method of Kunits [35]. Briefly, reaction mixtures (80 μl) contain 0.2% (w/v) casein in 50 mM Tris-HCl buffer (pH 8.0) and an appropriate amount of enzyme (final concentration of 0.001−0.02 mg/ml for proteases and 0.08 mg of total proteins per ml for crude extract were incubated at 37°C (proteinase K and trypsin), 70°C (papain), and 80°C (AWCE) for 90 min, 15 min, and 20 min, respectively. After incubation, reactions were terminated by the addition of 20 μl of 50% (w/v) trichloroacetic acid (TCA), followed by centrifugation at 15,000 × g for 10 min at 4°C. The absorbance of the resulting supernatant was measured at 280 nm. One unit (U) of protease activity was defined as the amount of enzyme that resulted in an increase in absorbance of 0.01 per min in the above conditions.

To compare the caseinolytic and keratinolytic activities of AWCE with those of other proteases, we also measured the increase in free amino acids as described previously [36]. Briefly, after enzymatic reactions were performed as described above, 150 μl of 3% ninhydrin solution and 150 μl of acetate-cyanide buffer (pH 5.2) were added to 30 μl of reaction mixture, boiled for 15 min for color development, and stopped by cooling on ice. After addition of 660 μl of isopropyl alcohol-water diluent, the absorbance was measured at 570 nm. One unit of protease activity was defined as the amount of enzyme that produced 1 nmole of free amino groups (equivalent to arginine) as products per min under the assay conditions. All measurements were performed in duplicate.

### Keratin hydrolysate preparation and LC-MS/MS analysis

After enzymatic reactions as described above, keratin hydrolysates were analyzed by reverse-phase HPLC-ESI-MS/MS using a Thermo (Dionex) UHPLC Ultimate 3000 directly connected to an AB SCIEX TripleTOF 5600+ mass spectrometer in direct injection mode as described previously [37]. Briefly, after a 10 μl of injection, keratin hydrolysates were loaded onto the ACQUITY UPLC BEH C_18_ column (2.1 × 50 mm, 1.7 μm BEH particle size; 130 Å pore size, Waters, Milford, MA) and eluted at a flow rate of 0.3 ml/min using the following gradient: mobile phase A (0.1% formic acid [v/v] in water), mobile phase B (0.1% formic acid [v/v] in acetonitrile); 1% solvent B (0−3 min), 1−50% solvent B (3−70 min), 50−100% solvent B (70−75 min), 100% solvent B (75−80 min), 100−1% solvent B in A (80−81 min) and at 99% solvent B (from 81−90 min), with a total runtime of 90 min including mobile phase equilibration.

MS analysis of peptide eluents was performed on a TripleTOF 5600 system (AB SCIEX, Concord, ON) fitted with a Nanospray III source and a pulled quartz tip as the emitter (New Objectives, Woburn, MA). Data were acquired using an ion spray voltage of 2.2 kV, curtain gas of 20 PSI, nebulizer gas of 6 PSI, and an interface heater temperature of 150°C. The MS was operated with a RP of 30,000 FWHM for TOF-MS scans. Advanced information-dependent acquisition (IDA) was used for MS/MS collection to obtain MS/MS spectra for 8, 20, or 50 product ions following each survey MS1 scan over a 250 ms acquisition time per MS/MS experiment. All ions selected for MS/MS had a 2+ or greater charge state. Four time bins were summed for each scan at a pulse frequency value of 11 kHz, through monitoring of the 40 GHz multichannel TDC detector with 4-anode/channel detection. A sweeping collision energy setting of 35 ± 15 eV was applied to all precursor ions for collision-induced dissociation. Dynamic exclusion was set for half the peak width (~8 s), and excluded precursors were placed on the exclusion list.

Data were processed using Protein PilotSoftware v. 4.0 (AB SCIEX, Foster City, CA) with the Paragon and Progroup Algorithm [38]. The software converts raw data (.wiff format) into peak lists (.mgf format) and re-calibrates data for searching (re-calibration of 20–25 ppm to re-tune the global dataset < 2 ppm). The FASTA database employed contained the *G*. *gallus* keratin sequence, as well as the *F*. *islandicum* AW-1 protease sequences, and this afforded the opportunity to employ the target decoy database search strategy [39]. Data containing both MS and MS/MS information were uploaded into PeakView software and used to generate MS-extracted ion chromatograms (XICs) for each identified peptide. The software algorithm simultaneously searched all modifications listed in UniMod (http://www.unimod.org/) [38] with a tolerance of ±0.05 Da for peptides and ±0.05 Da for MS/MS fragments. False discovery rate (FDR) analysis was also performed using integrated tools in ProteinPilot, which generated.mgf files that were subsequently searched against the current *G*. *gallus* keratins SwissProt database using the Mascot Server v. 2.2. For the Mascot search, carbamidomethyl (C) was set as a fixed modification and deamidation (N and Q) and oxidation (M) were set as variable modifications. The maximum missed cleavage = 2, peptide tolerance = ± 0.05 Da, and MS/MS tolerance = ± 0.03 Da.

## Results and discussion

### Characterization of the *F*. *islandicum* AW-1 crude extract (AWCE)

**Fig 1:**
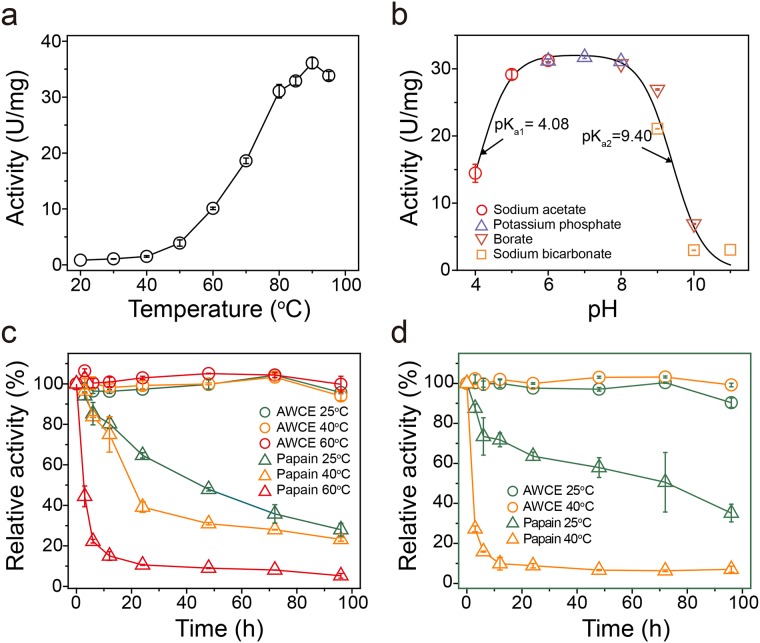
Physicochemical properties of crude extracts from *Fervidobacterium islandicum* AW-1 (AWCE). Effect of temperature (A) and pH (B) on the proteolytic activity of AWCE. (C) Time course of irreversible thermal inactivation of AWCE and papain at various temperatures. (D) Effect of 0.2% (w/v) SDS on the enzyme activity of AWCE and papain at various temperatures. After various periods of incubation at various temperatures, aliquots were withdrawn and their residual activities were measured under the standard assay conditions.

We previously isolated the native chicken feather-degrading bacterium *Fervidobacterium islandicum* AW-1 from an Indonesian hot spring, and this bacterium grew optimally at 70°C and was able to degrade native chicken feathers within 48 h under anaerobic conditions [14]. The recently reported near-complete genome sequence of *F*. *islandicum* AW-1 revealed that this bacterium possesses more than 50 different genes encoding proteases [23], suggesting that it may be a potent keratinase-producing organism. However, it remains unclear whether one or several proteases may be specific for feather keratin and hence involved in feather degradation. Given the absence of functional annotation of keratinases in *F*. *islandicum* AW-1, we first characterized the effect of temperature and pH on proteolytic activity using a crude extract (AWCE). As shown in Fig 1A and 1B, AWCE showed maximal proteolytic activity at around 90°C and was active between pH 6 and 8. The proteolytic activity of AWCE was retained for 96 h at 60°C and the half-life was 48 h at 80°C (Fig 1C). By comparison, the commercially available protease papain was readily inactivated and displayed a half-life of ~20 h, even at temperature as low as 40°C. Furthermore, the residual activity of the crude extract was retained without any loss of enzyme activity even after a 96 h-incubation at 40°C even in the presence of strong ionic detergents such as 0.25% SDS, whereas papain was significantly inactivated within half a day under the same conditions (Fig 1D). These results clearly indicated a robust proteolytic activity in AWCE that was highly thermostable, optimal around neutral pH, and resilient to detergents.

**Fig 2:**
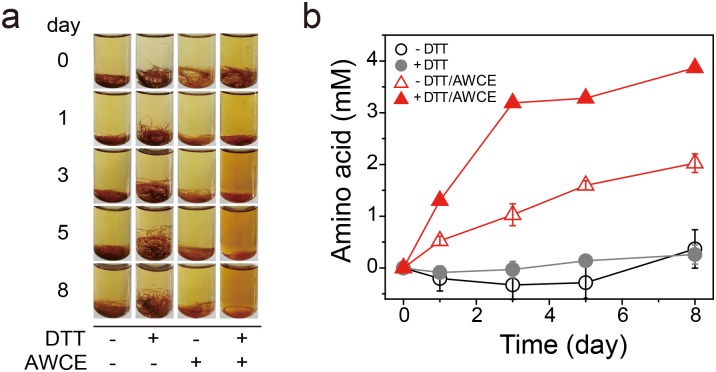
Keratinolytic activity of AWCE. Time course of decomposition of native chicken feathers by CEAW-1 at 80°C (A) and the release of free amino acids from feather hydrolysates (B) in the presence and absence of 10 mM DTT.

Next, we examined the feather-degrading activity of AWCE by incubating with 0.2% (w/v) native feather in the presence or absence of 10 mM dithiothreitol (DTT) under anaerobic conditions. As shown in Fig 2A, thermal incubation at 80°C resulted in minimal degradation of native feathers, regardless of the presence or absence of 10 mM DTT, whereas AWCE caused significant degradation of native feathers in the presence of DTT, although degradation was 2-fold lower in the absence of DTT (Fig 2B). This is presumably because breakage of intersubunit interactions in the form of disulfide bonds between cysteine residues that are rich in poultry feather keratin (i.e., sulphytolysis) is a prerequisite for the degradation of β-keratin [4]. Based on quantification of the amino acids released from feather hydrolysates, we concluded that subcellular fractions of *F*. *islandicum* AW-1 retained feather keratin-degrading activity, suggesting that AWCE contained proteases responsible for keratin degradation.

### Expression of recombinant keratin in *E*. *coli*

**Fig 3:**
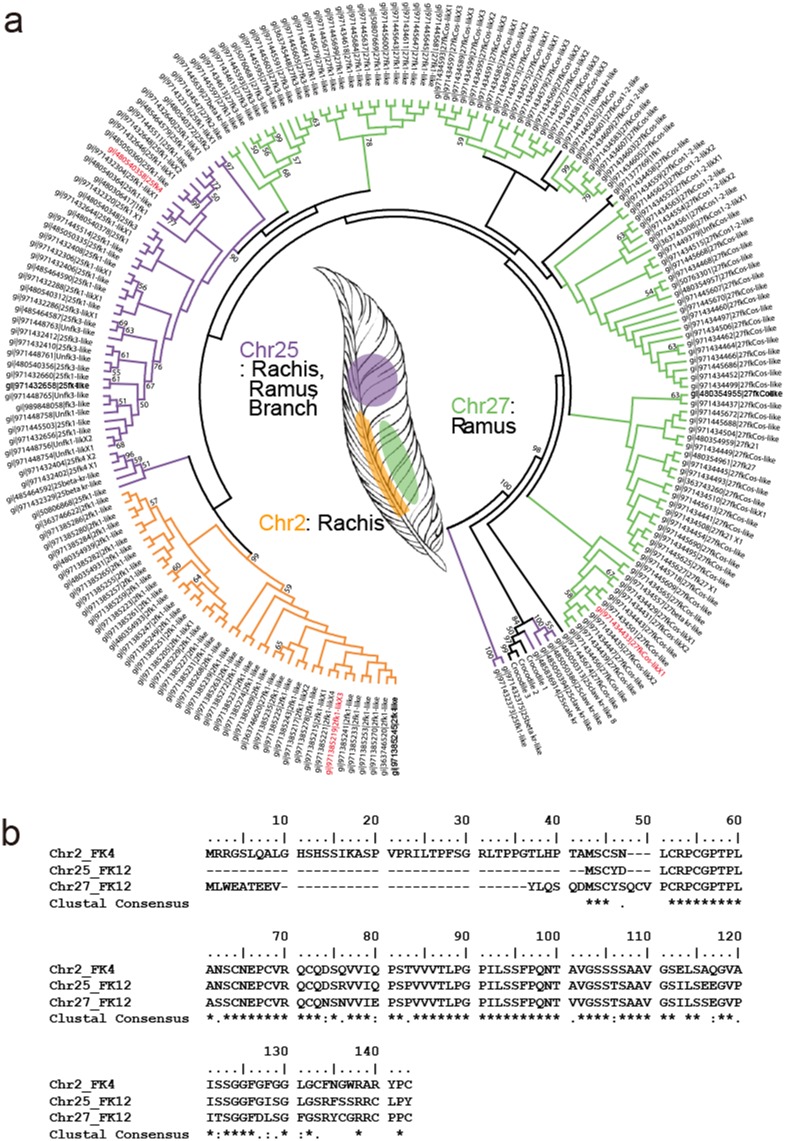
*Gallus gallus* feather keratins encoded on chromosomes 2, 25, and 27. (A) Consensus phylogenetic tree of β-keratin and related genes in *G*. *gallus*. Three β-keratin genes from crocodile are presented as outgroups with 204 β-keratin genes found in the chicken genome (*Gallus gallus* 5.0). Posterior bootstrapping provided statistical support for branches. The bootstrap values are listed for each major branch when they are above 50%. The feather β-keratin and feather-like β-keratin superfamilies encoded on chromosomes 2, 25, and 27 are colored orange, purple, and pale green, respectively. (B) Alignment of the amino acid sequences of Chr2_FK4 (NCBI accession no. gi|971385219), Chr25_FK12 (gi|480540358), and Chr27_FK12 (gi|971434433) β-keratins.

**Fig 4:**
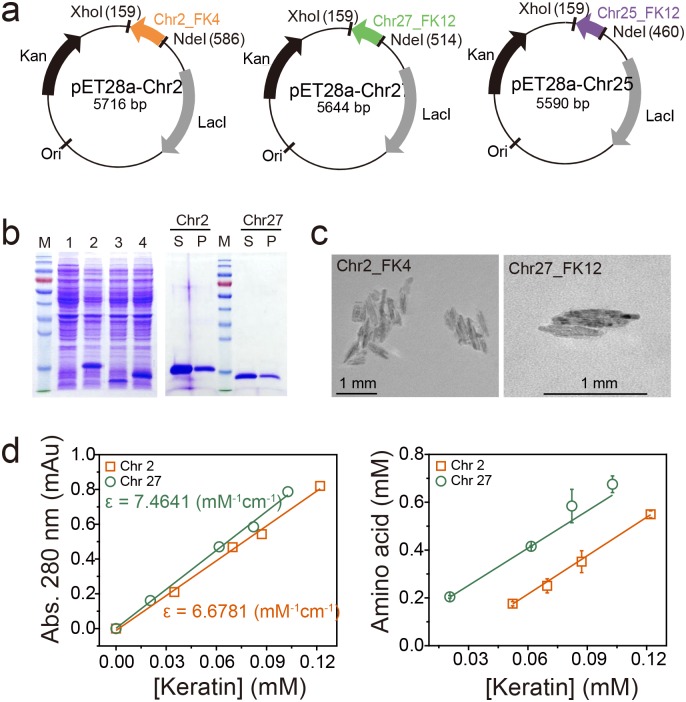
Expression and purification of soluble *G*. *gallus* β-keratins. (A) Construction of expression vectors for recombinant Chr2_FK4, Chr25_FK25, and Chr27_FK12 β-keratins. (B) SDS-PAGE analysis of recombinant keratins expressed in *E*. *coli*, and purification of soluble Chr2_FK4 and Chr27_FK12 β-keratins. Lane M, molecular weight markers; lane 1, *E*. *coli* BL21 (DE3); lane 2, *E*. *coli* BL21 (DE3) (pET-28a_Chr2); lane 3, *E*. *coli* BL21 (DE3) (pET-28a_Chr25); lane 4, *E*. *coli* BL21 (DE3) (pET-28a_Chr27); S, supernatant; P, pellet. (C) Transmission electron microscope images of recombinant β-keratins. (D) Quantification of soluble Chr2_FK4 and Chr27_FK12 β-keratins using Kunitz and ninhydrin assays. Linear correlation between the absorbance at 280 nm and the concentration of purified β-keratins.

A variety of proteases active against keratin derivatives have been identified and/or characterized (Table 1). Nevertheless, identification of proteases such as keratinases provides minimal information on their P1 and P1’ sites, or their substrate specificity. To the best of our knowledge, most keratinase activity assays have been performed using insoluble substrates such as azokeratin (similar to keratin azure), feather meal, and autoclaved chicken feathers (Table 1). These enzymatic assays are limited in their ability to quantify keratin degradation activity, and the results do not provide any real information on substrate specificity such as the residues preferentially bound in P1/P1’ sites. We attempted to rectify this in the present work by determining the keratinolytic activity of AWCE using a soluble intact poultry keratin as the substrate. To this end, we searched for chicken keratin sequences in appropriate databases using BLAST and surveyed previously published literature [28, 30]. We learned from the *Gallus gallus* 5.0 chromosome (Chr) sequences that there are putative feather keratin (FK)-encoding genes on Chr 2, 25, and 27. Alignment of the amino acid sequences of these putative keratins with 204 homologous keratin sequences was performed using Clustal W to yield a phylogenetic tree of feather keratin homologs (Fig 3A). Genes on Chr 2 and 25 are differentiated and are responsible for the rachis (i.e., as the central shaft) and barb (i.e., lateral branches off the rachis) structures of chicken feathers, respectively, whereas the gene encoded on Chr 27 is involved in the ramus as the central shaft of a barb [28], indicating that intensive β-keratin gene duplications on Chr 25 and 27 may contribute to increased differences in textures and rigidity of feather types. Thus, we chose Chr2_FK4, Chr25_FK12, and Chr27_FK12 as genes encoding potential keratin substrates and we chemically synthesized versions that were codon-optimized for expression in *E*. *coli* (Fig 3B). The synthesized genes were cloned into the pET-28a (+) expression vector and expressed under IPTG induction in *E*. *coli* BL21 (DE3). As shown in Fig 4A and 4B, all three synthesized genes were successfully overexpressed, albeit in insoluble inclusion body form. We therefore solubilized the inclusion bodies using 8 M urea, purified the denatured keratins by a Ni^2+^-NTA affinity chromatography, and refolded them using stepwise dialysis against buffer containing progressively less denaturant at room temperature (Fig 4B). We failed to obtain Chr25_FK12 in soluble form, but we successfully refolded Chr2_FK4 and Chr27_FK12 and obtained mg quantities from 1 l cultures as demonstrated by SDS-PAGE analysis (Fig 4B). Purified Chr2_FK4 and Chr27_FK12 displayed a linear relationship between the absorbance value at 280 nm and the protein concentration, and individual extinction coefficients were therefore measured (Fig 4D). Intriguingly, transmission electron microscopic analysis of their morphological characteristics revealed shorter pleated amyloid particles compared with those present in native chicken feathers (Fig 4C). The successful preparation of 2–3 mg of recombinant *G*. *gallus* keratins in soluble form provided ample material for subsequent keratinolytic activity assays.

### Keratinolytic activity of AWCE and various proteases towards soluble keratins

**Fig 5:**
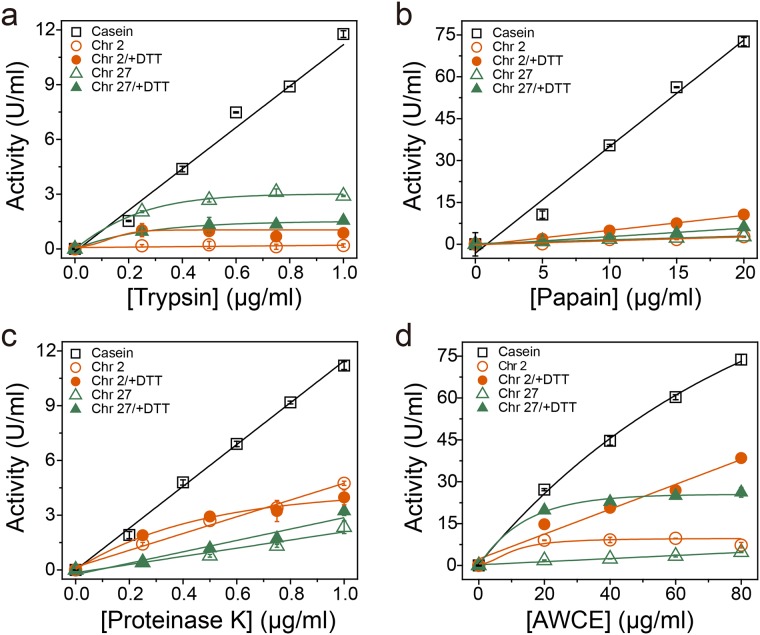
Comparison of the proteolytic activity of AWCE and other proteases towards casein and recombinant keratin substrates. Enzyme assays for each protease were performed under the standard assay conditions (see Materials and Methods).

To examine the keratinolytic activity of AWCE and other proteases using soluble keratins as substrates, we first compared their proteolytic activity with casein as a general substrate by measuring the release of free amino acids. As shown in Fig 5, the serine proteases trypsin and proteinase K, and the cysteine protease papain, displayed high proteolytic activity towards casein, with free amino acid liberation proportional to protein concentration, but these enzymes exhibited negligible activity towards soluble keratins. Indeed, trypsin and papain exhibited approximately 10-fold lower activity for both Chr2_FK4 and Chr27_FK12 than for casein, indicating that these proteases were not keratinases as expected (Fig 5A and 5B). By contrast, proteinase K, belonging to peptidase family S8, cleaves peptide bonds on the carboxyl site of aliphatic and aromatic amino acids with blocked α-amino groups, and it renowned for its broad substrate specificity [54]. Although proteinase K is not known as a keratinase, it did display a relatively weak but significant keratin-degrading activity (Fig 5C). However, AWCE exhibited a much stronger proteolytic activity towards casein, and a higher keratinolytic activity towards soluble keratins, as expected (Fig 5D). The keratinolytic activity of AWCE and other proteases towards soluble keratin substrates clearly showed a linear relationship between the protein concentration of and the concentration of free amino acids, as was the case with soluble casein as substrate.

**Fig 6:**
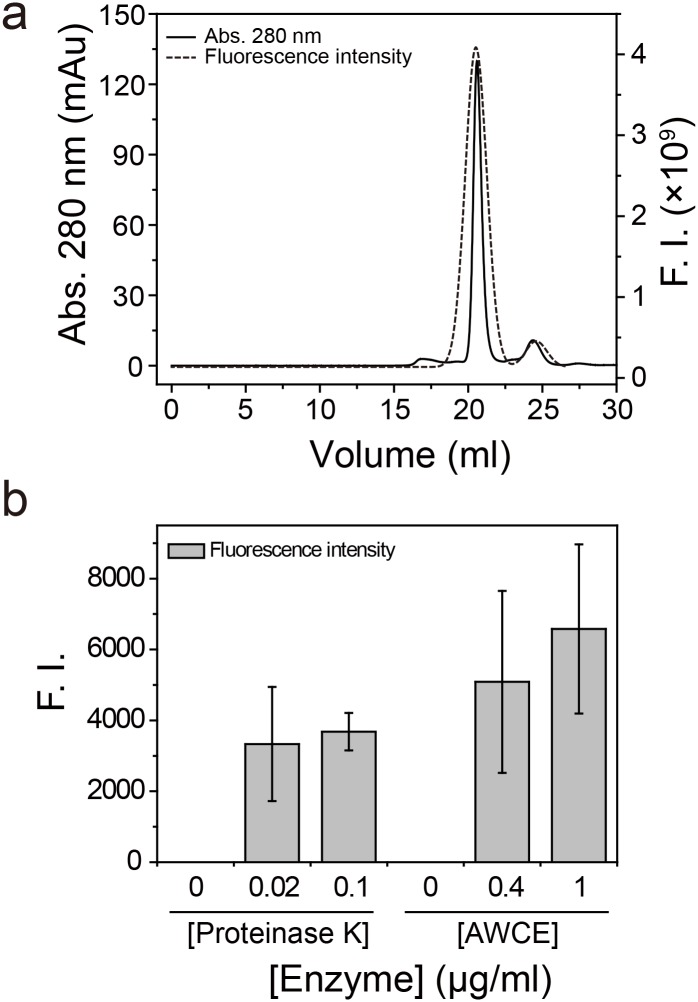
The degree of keratin degradation by AWCE. (A) Purification of fluorescein-5-maleimide (FM)-conjugated Chr27_FK12 β-keratin by Superdex 200 10/300 GL column chromatography. (B) Relative degradation of FM-conjugated Chr27_FK12 by AWCE and proteinase K.

Finally, to quantify the degree of keratin degradation, we first alkylated soluble keratin with FM in the presence of Tris (2-carboxyethyl) phosphine (TCEP) and purified the conjugate on a Superdex 200 10/300 GL column (Fig 6A). Thereafter, we analyzed the keratinolytic activity of AWCE and proteinase K with FM-labeled Chr27_FK12 as substrate. As shown in Fig 6B, thermal incubation at 80°C did not reveal a significant fluorescence intensity (FI) after TCA precipitation, suggesting that FM-labeled keratin was not degraded and was instead pelleted. However, incubation with proteinase K 37°C and AWCE at 80°C resulted in a large increase in the FI value of the supernatant after TCA precipitation of the reaction mixtures, indicating that proteinase K and AWCE exhibited keratin-specific proteolytic activity. We therefore concluded that the soluble keratins acted as suitable substrates in keratinase activity assays.

### Determination of the P1 sites for proteases in AWCE

**Fig 7:**
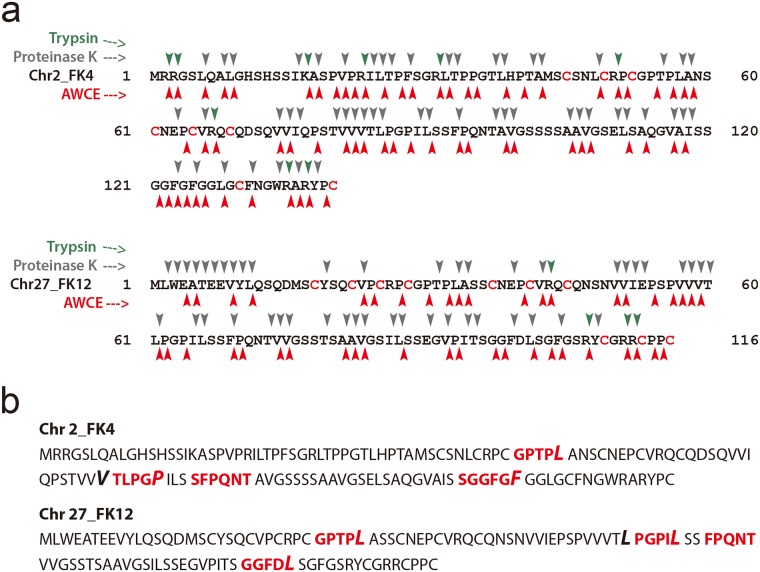
Keratin peptide mapping. (A) *In silico* digestion of soluble keratins by proteases and AWCE using the program PeptideCutter. (B) LC-MS/MS analysis of keratin hydrolysates generated by AWCE. Keratinolytic peptides matched with soluble Chr2_FK4 and Chr27_FK12 β-keratins are depicted in bold and colored red.

Based on our keratinolytic activity assay with native feathers and soluble keratins as substrates, we characterized the hydrolysates resulting from AWCE and proteinase K degradation using LC-MS/MS. For AWCE, we predicted the peptide mapping of keratin substrates using the Merops database (http://merops.sanger.ac.uk) [55]. As shown in Table 2, we first analyzed the predicted P1/P1’ sites of *F*. *islandicum* AW-1 proteases with putative proteases functionally annotated by their genome sequence [23], and the bioinformatics data were used to construct theoretical keratin hydrolysates generated by AWCE, using the PeptideCutter program (http://web.expasy.org/peptide_cutter/) as shown in Fig 7A. Accordingly, we performed *in silico* digestion of soluble keratins by proteases examined in this study, and enzymes specific for arginine and lysine at the P1 site, and phenylalanine and arginine at the P1' site, displayed little activity with keratins, except for proteinase K, which is known to have a broad substrate specificity [54, 56]. For instance, trypsin was expected to exhibit very poor activity for soluble keratin, consistent with its enzyme activity shown in Fig 5A. As expected, proteinase K exhibited a higher activity than trypsin towards soluble keratin, consistent with the *in silico* digestion (Fig 5C). On the basis of these results, we analyzed the keratin hydrolysates generated by both proteinase K and AWCE using the same LC-MS/MS approach. As shown in Fig 7B, all peptides hydrolyzed by each enzyme matched the keratin sequences derived from *G*. *gallus* sequences, indicating that soluble keratins can serve as substrates for screening and characterization of feather keratin-specific proteases as keratinases. Moreover, the identified peptide sequences provided important information on the residues that preferentially bind at the P1 and P1’ sites.

**Table 2:** Bioinformatic analysis of putative proteases from *F*. *islandiucm* AW-1 by MEROPS.

Gene name	Product (Annotation)	BLAST results	MEROPS ID	Cleavage site
NA23_06430	serine protease	Trypsin-like serine protease	S01.273	R or K/, V/
NA23_04280	peptidase M42	Glutamyl aminopeptidase/aminopeptidase 1	M42.001 M42.002	E/A (100%), D/A (74%)
NA23_07755	D-alanyl-D-alanine carboxypeptidase	D-alanyl-D-alanine carboxypeptidase VanY (family M15)	M15.010	D-Ala/D-Ala
NA23_01240	ATP-dependent Clp protease	Endopeptidase Clp(family S14)	S14.001	M/ALVP
NA23_10440	Peptidase M55	D-aminopeptidase DppA (family M55)	M55.001	D-Ala/D-Ala, D-Ala/Gly-Gly
NA23_05775	Peptidase S8	Peptidase S8_Thermitase like	S08.007	(A)AA/ or (A)AF/, F, A or L/
NA23_05565	Peptidase C15	Peptidase C15, Pyroglutamyl-peptidase type1	C15.001	Glp/
NA23_07735	Peptidase S9	Peptidase S9, Prolyl oligopeptidase family	S09.001	P/
NA23_04555	Signal peptidase 1	Signal peptidase 1 (LepB), family S26	S26.001	AXA/(P3, P1)
NA23_09915	Peptidase M23	Peptidase family M23	M23.001	G/G
NA23_06090	Peptidase S9	Peptidase S9, Prolyl oligopeptidase family	S09.001	P/
NA23_06420	Peptidase A24	Type Ⅳ prepilin peptidase type M1, family A24	A24.001	G/F
NA23_09700	Aminopeptidase	Glutamyl aminopeptidase/aminopeptidase 1	M42.001 M42.002	E/A (100%), D/A (74%)
NA23_08080	peptidase	isoaspartyl dipeptidase (metallo-type)	M38.001	Isoaspartyl/glycine (isoaspartyl dipeptides)

## Conclusions

In the present study, we characterized the keratinolytic ability of the feather-degrading bacterium *F*. *islandicum* AW-1. Several key experiments indicate that the complex supramolecular organization of feather keratins requires not only keratin-specific proteases for peptide degradation, but also several other functional enzymes involved in attachment to the surface of insoluble keratin polymers [20], deconstruction of the suprastructure [57], and breakage of intermolecular bonds, including disulfides via sulphytolysis [14, 17]. Together, these viewpoints support the notion that keratin degradation requires the synergistic action of sets of enzymes including keratinases, oxidoreductases, and cell wall-degrading glycosyltransferases (unpublished data). Therefore, to better understand the mechanistic features of feather degradation, identification of proteases that are highly specific for keratin is of the utmost importance. To this end, in the present work we attempted to remove some of the technical barriers that have prevented the development of keratinolytic enzyme assays based on soluble keratin substrates. Intriguingly, Yoshioka et al. [58] identified a *Bacillus* protein among more than 200 bacterial proteases that displayed keratinolytic activity, and the isolated enzyme exhibited a high capacity for degrading the scrapie form of the prion protein, PrP (Sc), as well as bovine spongiform encephalopathy-infected brain homogenates, suggesting that it could be used to the degrade pathogenic forms of prion proteins and other disease-associated protein aggregates. In this regard the soluble keratin-based assay system developed in the present study has great potential for characterizing proteases that may be of therapeutic use for treating disease-associated insoluble, misfolded, and aggregated proteins. The soluble keratins obtained in this study could provide reliable qualitative and quantitative information on the degradation of feather keratin, and could be used to characterize the substrate specificity of proteases more generally.
